# Galyl and Ludyl

**Published:** 1914-04

**Authors:** 


GALYL AND LUDYL. Troisfontaines, *Presse Med.,* November 1, 1913, reports favorable results with these new organic compounds of arsenic in syphilis.

